# Exploring Tumor Microenvironment in Breast Cancer: Parameters that can be Assessed with Light Microscopy

**DOI:** 10.5146/tjpath.2024.13748

**Published:** 2025-09-30

**Authors:** Seetu Palo

**Affiliations:** Department of Pathology and Laboratory Medicine, All India Institute of Medical Sciences, Hyderabad, India

Dear Editor,

The recently published study titled ‘Investigation of the Relationship Between Tumor Microenvironment and Prognostic Parameters in Invasive Breast Carcinomas of No Special Type: A Retrospective Analysis’ by Ozsen M et al addresses a relevant topic in predictive oncology, by investigating the tumor microenvironment (TME) and its relationship with prognostic parameters in invasive breast carcinoma (1). The TME comprises a wide array of inflammatory cells, cancer-associated fibroblasts, endothelial cells and pericytes, and other tissue-specific cell types like adipocytes and there is compelling evidence that they play a significant role in cancer development and progression as well as the response to chemotherapy, making them appealing prognostic biomarkers and therapeutic targets (2).

The study’s strength lies in its thorough evaluation of several aspects of TME, including stromal tumor-infiltrating lymphocytes (TIL), stromal reactions, tumor budding, and growth patterns in a relatively large cohort of 271 cases. While the study adheres to the WHO guidelines for TIL evaluation, other parameters like stromal reactions and tumor budding are evaluated based on subjective criteria that need further refinement from the authors to enhance reproducibility. For example, both peri- and intra-tumoral stromal reaction intensity is categorized into broad groups (absent, mild, moderate, prominent), but the criteria for these distinctions are not clearly defined and could lead to intra- and inter- observer variability. Similarly, although the authors have defined tumor budding as ‘isolated tumor cells or groups of less than five tumor cells’, evaluating tumor budding in a semi-quantitative manner, rather than the dichotomous evaluation as ‘present’ or ‘absent’, would have enhanced the study’s finding in this context. Also, tumor budding is typically assessed at the invasive tumor front and hence the authors could shed more light upon their experience in assessing it in the intra-tumoral area (3). Furthermore, tumor budding and tumor growth pattern are characteristics of the tumor itself rather than the host microenvironment. The study recorded no statistically significant relationship between the studied TME parameters and recurrence-free or overall survival period. A brief note on the whether any adjuvant chemotherapy and/or radiotherapy was received by the patients included in the study cohort would help in better interpretation of this result of the study.

Overall, the study is well-designed with apt inclusion and exclusion criteria and makes a valuable contribution to the ongoing research on TME in breast cancer. As the authors have acknowledged, it is advantageous that these parameters, namely, stromal reactions, tumor budding, and growth patterns can be easily estimated using H&E-stained slides without incurring extra costs or time delays. Apart from the TIL that comprises the mononuclear inflammatory cells, infiltrating stromal neutrophils (tumor associated neutrophils) and eosinophils (tumor associated tissue eosinophilia) can also be assessed using H&E-stained slides and are of prognostic and predictive relevance in breast cancer (4,5).

## Conflict of Interest

The authors declare that they have no conflict of interest.

## References

[ref-1] Ozsen Mine, Tolunay Sahsine, Senol Kazim, Deligonul Adem, Gokgoz Sehsuvar, Evrensel Turkkan (2024). Investigation of the Relationship Between Tumor Microenvironment and Prognostic Parameters in Invasive Breast Carcinomas of No Special Type: A Retrospective Analysis. Turk Patoloji Derg.

[ref-2] Ji Fei, Yuan Jiao-Mei, Gao Hong-Fei, Xu Ai-Qi, Yang Zheng, Yang Ci-Qiu, Zhang Liu-Lu, Yang Mei, Li Jie-Qing, Zhu Teng, Cheng Min-Yi, Wu Si-Yan, Wang Kun (2021). Tumor Microenvironment Characterization in Breast Cancer Identifies Prognostic and Neoadjuvant Chemotherapy Relevant Signatures. Front Mol Biosci.

[ref-3] Kundu Tamanna, Giri Ranjana, Hota Subhransu K., Sahu Subrat K., Senapati Urmila (2023). Tumour budding in invasive breast carcinoma and its correlation with histopathological prognostic parameters and hormone receptor status. J Cancer Res Ther.

[ref-4] Gong Yi-Ting, Zhang Li-Jun, Liu Yi-Chen, Tang Min, Lin Jia-Yi, Chen Xin-Yi, Chen Yi-Xu, Yan Yue, Zhang Wei-Dong, Jin Jin-Mei, Luan Xin (2023). Neutrophils as potential therapeutic targets for breast cancer. Pharmacol Res.

[ref-5] Poncin Aurélie, Onesti Concetta Elisa, Josse Claire, Boulet Delphine, Thiry Jérôme, Bours Vincent, Jerusalem Guy (2021). Immunity and Breast Cancer: Focus on Eosinophils. Biomedicines.

